# Supplementary material for: Transcriptional Up-Regulation of FBXW7 by KCa1.1 K+ Channel Inhibition through the Nrf2 Signaling Pathway in Human Prostate Cancer LNCaP Cell Spheroid Model
Source: Int J Mol Sci. 2024 May 30;25(11):6019. doi: 10.3390/ijms25116019 (PMC11172474; doi:10.3390/ijms25116019)
Supplement: Supplementary file 1 [file ijms-25-06019-s001.zip › ijms-2986819-supplementary.pdf]

Supplementary Materials

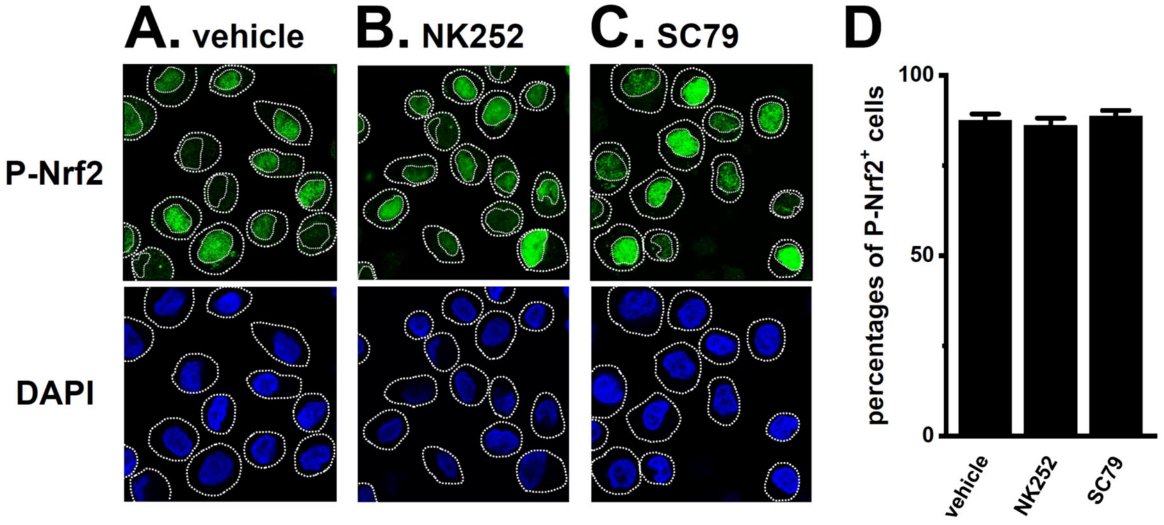

Figure S1. Effects of the treatment with the Nrf2 activator, NK252 and the Akt activator, SC79 on the nuclear translocation of P-Nrf2 in isolated cells from the LNCaP spheroid model. A-C: Confocal fluorescent images of Alexa Fluor 488-labeled P-Nrf2 (green, upper panels) in vehicle- (A), 100 μM NK252- (B), and 10 μM SC79-treated (C) LNCaP cells. Nuclear morphologies were assessed using DAPI staining (blue, lower panels). Dashed lines show the nuclear and cell boundaries. D: Summarized results of the percentages of P-Nrf2-positive cells in LNCaP cells (n = 6 for each, more than 30 cells for each data point).

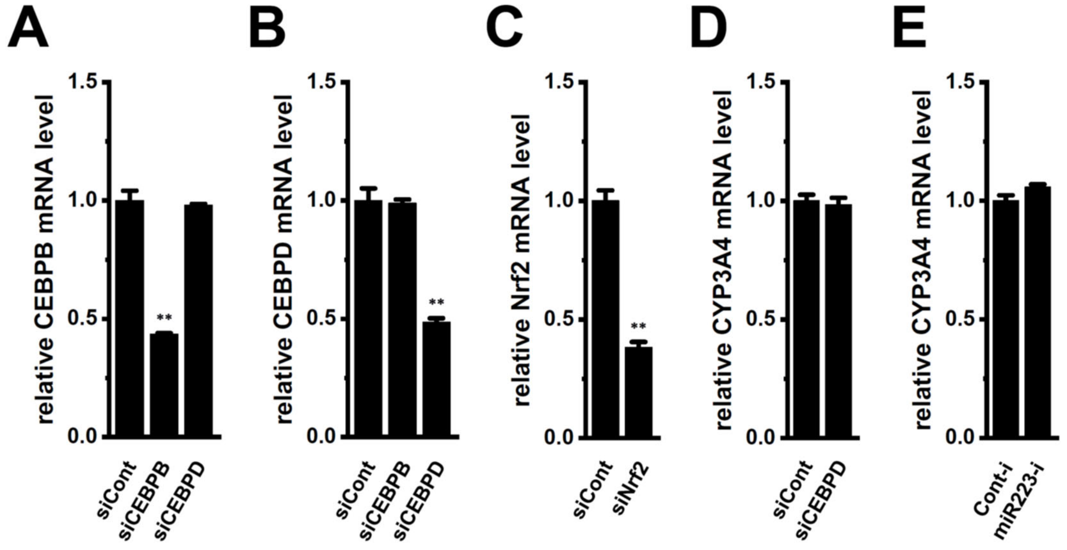

Figure S2. The transcriptional repression efficacy of CEBPB siRNA (siCEBPB), CEBPD siRNA (siCEBPD), and Nrf2 siRNA (siNrf2) and effects of the transfection of CEBPD siRNA and miR223 inhibitor on expression levels of CYP3A4 transcripts in the LNCaP spheroid model. A-C: Real-time PCR of CEBPB (A), CEBPD (B), and Nrf2 (C) transcripts in the LNCaP spheroid model transfected with negative control siRNA (siCont), siCEBPB, siCEBPD, and siNrf2 for 72 hr (n = 4 for each). After normalization to ACTB mRNA expression levels, the mRNA expression levels in siCont were expressed as 1.0 (n = 4 for each). D,E: Real-time PCR of CYP3A4 in the LNCaP spheroid models transfected with siCont, siCEBPD, miRNA inhibitor negative control (Cont-i), and miR223 inhibitor (miR223-i). After normalization to ACTB mRNA expression levels, CYP3A4 mRNA expression levels in the siCont- and Cont-i-transfected groups were expressed as 1.0 (n = 4). **: $P < 0.01$ vs. siCont.

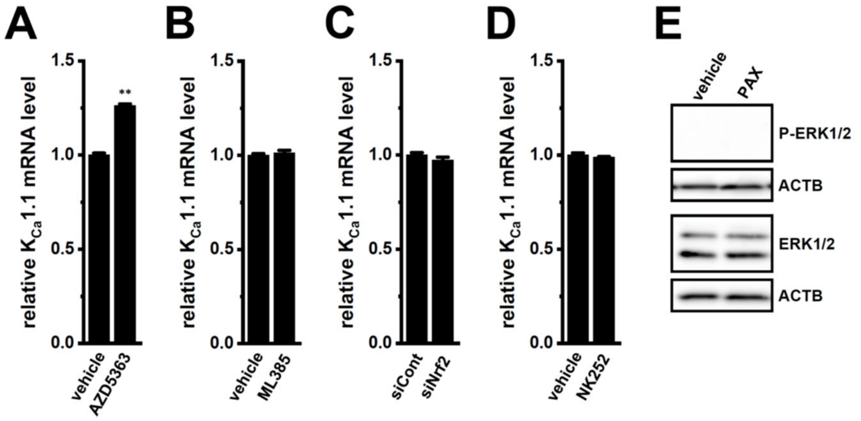

Figure S3. Effects of the treatment with the Akt inhibitor, AZD5363, the Nrf2 inhibitor, ML385, and the Nrf2 activator, NK252, and the transfection of siNrf2 on expression levels of K_{Ca}1.1 transcripts and effects of the pharmacological inhibition of K_{Ca}1.1 with PAX for 2 hr on expression levels of P-ERK1/2 proteins in the LNCaP spheroid model. A-D: Real-time PCR of K_{Ca}1.1 in the LNCaP spheroid models treated with 2 μM AZD5363, 10 μM ML385, and 100 μM NK252 and transfected with control siRNA (siCont) and Nrf2 siRNA (siNrf2). After normalization to ACTB mRNA expression levels, K_{Ca}1.1 mRNA expression levels in the vehicle control-treated (A,B,D) and siCont (C)-transfected groups were expressed as 1.0 (n = 4). E: Western blot showing P-ERK1/2 and total ERK1/2 (ERK1/2) in the vehicle- and 10 μM PAX-treated LNCaP spheroid models for 2 hr. Blots were probed with anti-P-ERK1/2, anti-ERK1/2 (approx. 40 kDa), and anti-ACTB (approx. 45 kDa) antibodies. **: $P < 0.01$ vs. the vehicle control.

Table S1. PCR primer information for real-time PCR.

Target name	Genbank accession number	Primer sequence		amplicon length (bp)
		Forward (5' to 3')	Reverse (5' to 3')	
FBXW7	NM_033632	TCACAAATGAGAGACAACATCATCA	ACAACGCACAGTGGAAGTATGC	120
K_{Ca}1.1	NM_001014797	CCATTTGGTGGAGAATTCAGG	ACATCCCCATAACCAACGGTG	120
CEBPB	NM_001285878	GCCCTCGCAGGTCAAGAG	TGCGCACGGCGATGT	107
CEBPD	NM_005195	TCCTGTTGATGCAGCTAAGGTACA	GCATGCTCAGTCTTTTCCTCTTATC	120
Nanog	NM_024865	CCTTCCTCCATGGATCTGCTTA	CTTGACCGGGACCTTGTGTTC	120
c-Myc	NM_002467	GCTCCTGGCAAAAGGTCAGA	CGCTGCGTAGTTGTGCTGAT	120
ACTB	NM_001101	AGGCCAACCGCGAGAAGATG	GCCAGAGGCGTACAGGGATA	101
miR135a-5p sense	Ref. [38]	CGCAGTATGGCTTTTTATTCCT	mRQ 3'Primer	
miR223-5p sense	Ref. [38]	CGCAGTGTCAGTTTGTCA	mRQ 3'Primer	
U6	NR_004394	CTCGCTTCGGCAGCACA	AACGCTTCACGAATTTGCGT	94
